# Optimal Management of Genetic Diversity in Subdivided Populations

**DOI:** 10.3389/fgene.2019.00843

**Published:** 2019-09-13

**Authors:** Eugenio López-Cortegano, Ramón Pouso, Adriana Labrador, Andrés Pérez-Figueroa, Jesús Fernández, Armando Caballero

**Affiliations:** ^1^Departamento de Bioquímica, Genética e Inmunología, Universidade de Vigo, Vigo, Spain; ^2^Centro de Investigación Marina (CIM-UVIGO), Universidade de Vigo, Vigo, Spain; ^3^Departamento de Mejora Genética, Instituto de Investigación y Tecnología Agraria y Alimentaria (INIA), Madrid, Spain

**Keywords:** conservation genetics, population management, allelic diversity, heterozygosity, genetic markers, SNP, haplotypes

## Abstract

One of the main objectives of conservation programs is the maintenance of genetic diversity because this provides the adaptive potential of populations to face new environmental challenges. Genetic diversity is generally assessed by means of neutral molecular markers, and it is usually quantified by the expected heterozygosity under Hardy-Weinberg equilibrium and the number of alleles per locus or allelic diversity. These two measures of genetic diversity are complementary because whereas the former is directly related to genetic variance for quantitative traits and, therefore, to the short-term response to selection and adaptation, the latter is more sensitive to population bottlenecks and relates more strongly to the long-term capacity of adaptation of populations. In the context of structured populations undergoing conservation programs, it is important to decide the optimum management strategy to preserve as much genetic diversity as possible while avoiding inbreeding. Here we examine, through computer simulations, the consequences of choosing a conservation strategy based on maximizing either heterozygosity or allelic diversity of single-nucleotide polymorphism haplotypes in a subdivided population. Our results suggest that maximization of allelic diversity can be more efficient in maintaining the genetic diversity of subdivided populations than maximization of expected heterozygosity because the former maintains a larger number of alleles while making a better control of inbreeding. Thus, maximization of allelic diversity should be a recommended strategy in conservation programs for structured populations.

## Introduction

Genetic diversity is the fuel for the adaptation of species to the environmental challenges and one of the main control variables to be assessed within the planetary boundaries framework (Steffen et al., 2015). Conservation of genetic diversity is also one of the main objectives for guaranteeing the long-term survival of species or breeds at risk of extinction (Frankham et al., 2010; Allendorf et al., 2013; Oldenbroek, 2017). Genetic diversity is generally assessed by means of neutral molecular markers in population genetics and conservation biology studies (Toro et al., 2009; Kirk and Freeland, 2011; Allendorf et al., 2013), and it is usually measured by the expected heterozygosity under Hardy-Weinberg equilibrium (Nei, 1973) and by the number of alleles per locus for multiallelic markers or allelic diversity. These two measures of genetic diversity are complementary because whereas the former is directly related to genetic variance for quantitative traits and, therefore, to the short-term response to selection and adaptation for these traits (Falconer and Mackay, 1996), the latter is more sensitive to population bottlenecks (Luikart et al., 1998; Leberg, 2002), being thus useful to monitor them, and relates more strongly to the long-term response to natural and artificial selection (James, 1970; Hill and Rasbash, 1986; Wilson et al., 2009; Medugorac et al., 2011; Caballero and García-Dorado, 2013; Vilas et al., 2015). In the case of structured populations, subpopulation differentiation is traditionally measured through differences in gene frequency of alleles (Wright, 1952), but alternative ways to measure it based on allelic diversity have also been proposed (Petit et al., 1998; Jost, 2008; Caballero and Rodríguez-Ramilo, 2010; Jost et al., 2017).

The consensus criterion for the maintenance of genetic diversity in conservation and animal breeding programs is the maximization of expected heterozygosity, which is equivalent to the minimization of mean weighted coancestry (Toro and Pérez-Enciso, 1990; Ballou and Lacy, 1995; Meuwissen, 2007) and implies the maximization of effective population size (Caballero and Toro, 2000, Caballero and Toro, 2002). However, allelic diversity has also been proposed to establish conservation priorities (Bataillon et al., 1996; Petit et al., 1998; Fernández et al., 2004; Simianer, 2005; Caballero and Rodríguez-Ramilo, 2010; Medugorac et al., 2011; Jost et al., 2017; Ramljak et al., 2018; Zhang et al., 2018), and there is an increasing number of methods and computer tools developed to estimate and predict allelic richness (Belkhir et al., 2006; Szpiech et al., 2008; Bashalkhanov et al., 2009) and to retain the largest allelic diversity in conservation programs (Fernández et al., 2004; Weiser et al., 2012; López-Cortegano et al., 2019). Microsatellite analysis has also revealed that allelic richness is a better proxy for genome-wide single-nucleotide polymorphism (SNP) diversity than expected heterozygosity (Fischer et al., 2017). In addition, it has been argued that the number of allelic variants after a bottleneck might be the main factor responsible for the response to long-term selection and selection limits (p. 289) (Allendorf et al., 2013). In fact, Vilas et al. (2015) showed through experimental studies and simulation analyses that the long-term adaptive potential of a population is better indicated by allelic diversity than by expected heterozygosity.

Conservation programs can be aimed at maximizing either expected heterozygosity or allelic diversity. Fernández et al. (2004) compared these two alternative strategies for a single undivided population. In a set of simulations, populations were maintained over generations by choosing the parents’ contributions to progeny that maximize the expected heterozygosity for multiallelic genetic markers. In another set, contributions were sought to maximize the number of marker alleles in the progeny. The results showed that each maximization method was, as would be expected, more efficient in maintaining each corresponding diversity measure. However, maximization of heterozygosity was able to maintain levels of allelic diversity almost as high as the method specifically devoted to that task. The explanation was that maximization of heterozygosity leads marker alleles toward intermediate frequencies because the maximal heterozygosity occurs when alleles are at equal frequencies. Thus, by spreading rare alleles to intermediate frequencies, their chances of loss by genetic drift are reduced. A method specifically focused on keeping allelic diversity was effective in doing so but some rare alleles were maintained at low frequencies, being more likely to be eventually lost.

The results from Fernández et al. (2004) were carried out in the context of a single undivided population. Most populations, however, in nature and in conservation programs (zoos, germplasm collections, botanic gardens, etc.) are subdivided. As suggested by preliminary analyses, the outcomes of maximizing expected heterozygosity or allelic diversity could be very different in subdivided populations (López-Cortegano et al., 2019). Thus, a question arises as to which of these methods is more efficient in maintaining genetic diversity while controlling inbreeding in structured populations. Here, we address this issue by performing simulations of a subdivided population and a conservation program where maximization of heterozygosity and allelic diversity are carried out for two sets of genetic markers, one representing a small number of known loci where diversity should be preserved and another aimed to perform whole-genome management. Because of the increasing availability of genotyping and sequencing projects, we focus on haplotypic combinations of SNPs as the marker of choice for future conservation strategies.

## Materials and Methods

**Figure 1:**
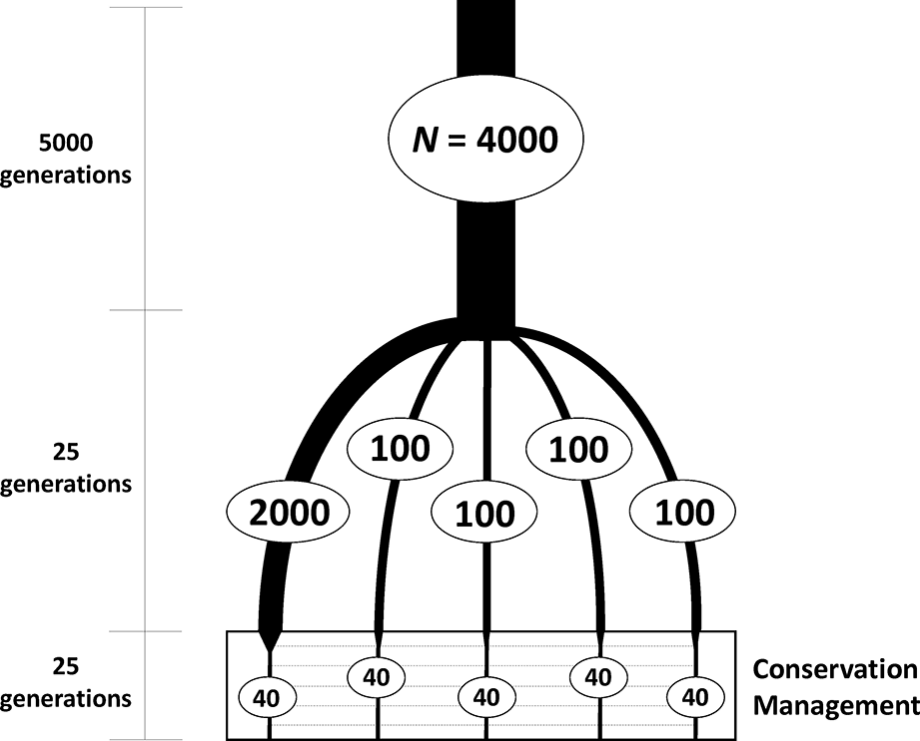
Scheme of the evolutionary history of the simulated subdivided population to be used as a base for a conservation program. One ancestral population of large size (*N* = 4,000 diploid individuals) was first maintained for a long period of time to reach mutation-drift equilibrium. From this ancestral population, five subpopulations (with constant population sizes as shown in the figure) were founded and maintained independently for 25 generations. These subpopulations were thereafter maintained with 40 individuals each and subjected to a conservation program aimed at maximizing either expected heterozygosity or allelic diversity of neutral markers, with some migration allowed between subpopulations.

Simulations were carried out in two steps. In the first, individual-based forward simulations were run to generate a subdivided population (**Figure 1**). An ancestral large population of 4,000 individuals was first run for 5,000 generations to build sufficient neutral genetic variation under a mutation-drift equilibrium. From this large base population, five subpopulations were founded, one of size *N* = 2,000 and four of size *N* = 100 individuals, to obtain different degrees of variation within subpopulations, which were maintained independently for 25 generations of random mating. The software SLiM 3 (Haller and Messer, 2018) was used as a forward genomic simulator in this first step. All simulations involved a sequence of 10 Kb with mutation rate of 5 × 10^-5^ per nucleotide and generation and a recombination rate of 10^-6^ between consecutive nucleotides in the formation of gametes. These mutation and recombination rate values were chosen to obtain a sufficiently high number and density of polymorphic loci within the simulated sequence. Additional simulations were also performed assuming a recombination rate one order of magnitude higher. Random mating of parents under the assumption of neutrality was implemented.

The second step of the study was the conservation management of the structured population created from the previous simulation. From each of the five subpopulations, a sample of *N* = 40 individuals (20 of each sex) was obtained and maintained with that size under a common conservation scheme based on maximization of either expected heterozygosity or allelic diversity for 25 generations with controlled migration between subpopulations. Marker loci to be used for analysis and management were assumed to be haplotypes of groups of five consecutive SNPs, such that the different haplotypic combinations of SNPs per locus were considered as different alleles, providing a maximum of 32 per locus. The total number of available loci was about 2,000, but the number of segregating loci available for analysis at the start of the conservation management process was approximately 1,200.

For conservation management, we used the software Metapop2 (López-Cortegano et al., 2019), which provides in each generation the optimal mating crosses and contributions from parents to the next generation as well as the number and specific migrants across subpopulations to maximize either heterozygosity or allelic diversity with a control in the number of migrations. With this program, total heterozygosity (*H* *_T_*) is partitioned, following Nei (1973), in the average expected heterozygosity within subpopulations assuming Hardy-Weinberg proportions (*H* *_S_*) and the average Nei’s minimum genetic distance between subpopulations, averaged over all possible pairs of subpopulations (*D* *_G_*). In an analogous way, allelic diversity is partitioned, following Caballero and Rodríguez-Ramilo (2010), in a within- and between-subpopulation component of variation. The within-subpopulation component is the average number of alleles segregating in the subpopulations minus one (*A* *_S_*). The between-subpopulation component (*D* *_A_*) is calculated as the number of alleles present in a subpopulation and absent in other when subpopulations are compared in pairs and averaged over all possible pairs of subpopulations. Total allelic diversity is then defined as *A* *_T_* = *A* *_S_* + *D* *_A_* and represents the total number of alleles present in a given pair of subpopulations, averaged for all possible pairs.

The Metapop2 software performs an optimization of contributions of parents to progeny and migrations between subpopulations with the dynamic method of Fernández et al. (2008) to maximize diversity. Maximization of total expected heterozygosity (maxH_T_) or total allelic diversity (maxA_T_) is obtained by maximizing the functions *H* *_T_* = *D* *_G_* + *λH* *_S_* and *A* *_T_* = *D* *_A_* + *λA* *_S_*, respectively, where *λ* is the desired weight given to the within-subpopulation component. In addition, the program also maximizes the total allelic number in the whole population (maxK) by managing contributions from parents to progeny and migrations so that the global probability of alleles’ losses in the progeny is minimized (Vales-Alonso et al., 2003; Fernández et al., 2004). Note that maxK pursues a maximization of the total number of alleles in the population without regard to the distribution of these across subpopulations. Because a maximum number of alleles in the whole population would be obtained with a maximum differentiation between subpopulations, maxK is expected to lead to such a situation. Maximization of *A* *_T_*, in contrast, implies a control on the distribution of the alleles maintained across subpopulations particularly if different weights are given to the within- and between-subpopulation components of diversity. Thus, alleles can be conserved uniformly distributed, leading to a reduction in the differentiation between subpopulations, or variably distributed across subpopulations, leading to an increase in the differentiation (López-Cortegano et al., 2019). At one extreme, each allele of a locus could be maintained in a different subpopulation. At the other, all different alleles for a locus could be maintained simultaneously in all subpopulations.

Management was run assuming two different objectives: (1) Conservation of diversity for a particular set of loci for which one locus every 100 in the genome was used for management and genetic variation was measured directly on that set of loci. This refers to a scenario in which a few known loci or genomic regions of particular interest have to be managed, for example, for loci that are known to have an effect on a particular trait of interest, such as those affecting a productive trait, the immune system, and so on. (2) Conservation of diversity in the whole genome for which one locus every 10 was used for management and the results were analyzed for all genomic loci. This is a situation where a number of markers are used for conserving overall genetic diversity. For this latter case, we used a modification of the software Metapop2 (López-Cortegano et al., 2019). With the assumed simulated sequence length and recombination rates, the density of markers would be in the range between 1,200 and 12,000 per Morgan, thus implying a high marker density in prevision of the increasing availability of dense SNP chips for more and more species. In the management period, it was assumed that there was no recombination within loci (*i.e.,* between SNPs of a particular haplotype) and recombination was free between them, which are reasonable assumptions given the short number of SNPs per locus and the scarcity of loci used along the sequence.

The optimization was carried out for 25 generations generally assuming a value of *λ* = 1, thus giving the same weight to within- and between-subpopulation components. However, other values of *λ* were also considered, including those for which all weight is given to between-subpopulation diversity (ခ*λ* = 0), all weight is given to within-subpopulation diversity (␭*λ* = 1000), and *λ* = 0.5, a value suggested to maximize the total genetic variance of a hypothetical quantitative trait (Bennewitz and Meuwissen, 2006). A maximum possible number of migrants of one per subpopulation and generation were assumed, a typical rule of thumb suggested to maintain a considerable differentiation between subpopulations but avoiding an excessive increase in inbreeding (Mills and Allendorf, 1996). In all cases, 10 replicates of the base population were simulated and, for each of them, 10 different sampling events and management processes were run. In every generation, the average values over replicates of expected heterozygosity measures (*H* *_T_*, *H* *_S_* and *D* *_G_*), allelic diversity measures (*A* *_T_*, *A* *_S_*, *D* *_A_* and *K*), and the observed marker homozygosity of all individuals in the subpopulations (to which we will refer to as molecular inbreeding, *F*, and which includes homozygotes identical by descent and identical in state) were obtained from the 12 managed markers for the scenario aimed at conserving diversity for a specific set of loci and from the whole sequence in the scenario aimed at conserving diversity for the whole genome.

## Results

**Figure 2:**
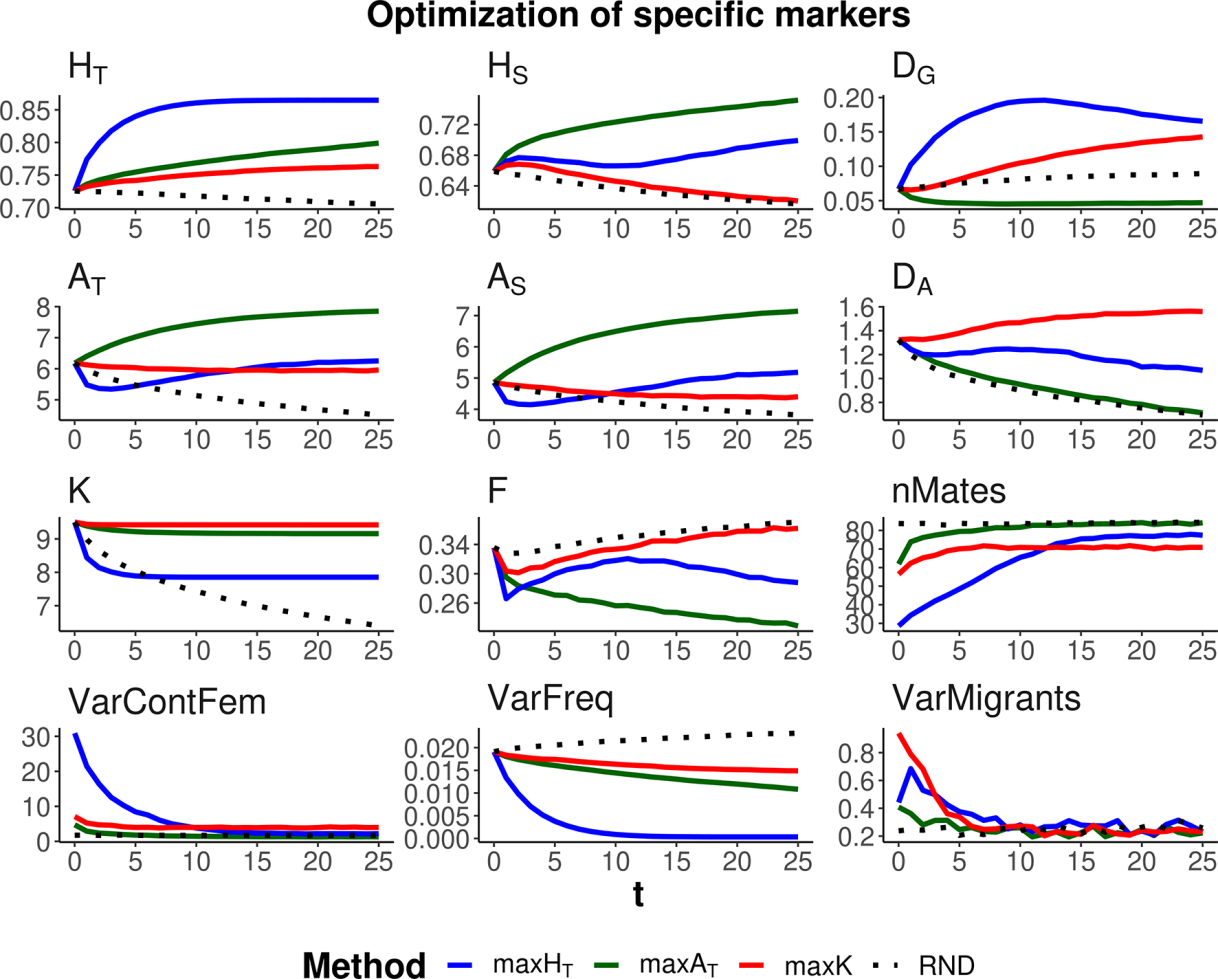
Changes in different diversity parameters over generations (*t*) in a subdivided population subjected to three optimization methods, maxH_T_ (blue line), maxA_T_ (green line), and maxK (red line), and an unmanaged control (RND, dotted black line). Optimization was made for a particular set of loci (12 multiallelic single-nucleotide polymorphism haplotype markers). In the case of maxH_T_ and maxA_T_, a between-population weighting factor of *λ* = 1 (*i.e*.*,* equal weight for within- and between-subpopulation components of diversity) was assumed. One migrant per subpopulation and generation was considered in the optimizations. Statistics measured in the managed markers: total heterozygosity (*H* *_T_*) and its within- and between-subpopulation components (*H* *_S_* and *D* *_G_*); total allelic diversity (*A* *_T_*) and its within- and between-subpopulation components (*A* *_S_* and *D* *_A_*); total number of alleles in the population (*K*); average coefficient of molecular inbreeding of individuals (*F*); number of pairing mates involved in the different procedures (nMates); variance of the contribution from female parents to progeny (varContFem); variance of allelic frequencies with loci (VarFreq); and variance of the number of migrants per subpopulation (VarMigrants). Standard errors for means are lower than 0.01 (allelic measures), 0.001 (heterozygosity measures), and 0.002 (*F*).

**Figure 3:**
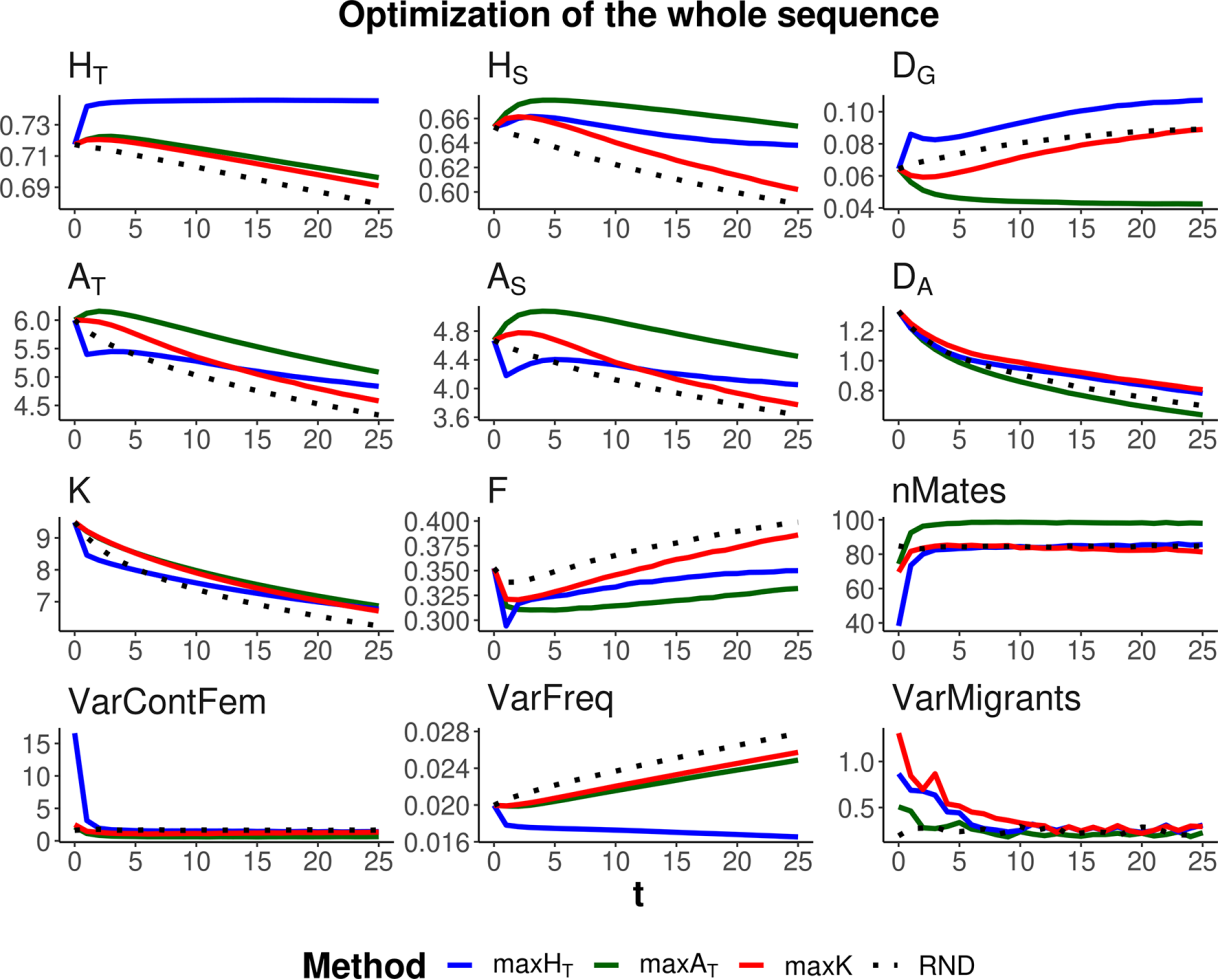
Changes in different diversity parameters over generations (*t*) in a subdivided population subjected to three optimization methods, maxH_T_ (blue line), maxA_T_ (green line), and maxK (red line), and an unmanaged control (RND, dotted black line). Optimization was made for 120 multiallelic (single-nucleotide polymorphism haplotype) markers, but statistics were calculated for the whole simulated genome. Simulation characteristics and statistics as in **Figure 2**. Standard errors for means are lower than 0.01 (allelic measures), 0.001 (heterozygosity measures), and 0.002 (*F*).

Three optimization methods were compared (maxH_T_, maxA_T_, and maxK), aimed at maximizing global heterozygosity *H* *_T_*, global allelic diversity *A* *_T_*, and the total number of alleles *K*, respectively. The evolution of these parameters and their within- and between-subpopulation components are shown in **Figures 2** and **3** when the same weight is given to within- and between-subpopulation diversity (*λ* = 1). As expected, no management (RND; black dotted lines) led to a generalized loss of genetic diversity and to an increase in molecular inbreeding whereas any of the specific management methods increased diversity or restrained its loss through generations. The relative performance of the different optimization methods was very similar for scenarios aiming at the conservation of diversity for either a particular set of loci (**Figure 2**) or the whole genome (**Figure 3**). Thus, we describe them simultaneously.

As expected, each maximization method maintained higher levels of the corresponding measure of diversity. Thus, maxH_T_ (blue lines) was the best method, preserving *H* *_T_* in the global population by means of an initial increase in the diversity between subpopulations (*D* *_G_*) while keeping or slightly decreasing that within subpopulations (*H* *_S_*). Method maxA_T_ (green lines) produced the largest *A* *_T_* by increasing or keeping a high diversity within subpopulations (*A* *_S_*) and decreasing that between subpopulations (*D* *_A_*). Finally, maxK (red lines) maintained the largest number of alleles segregating in the whole population (*K*), although maxA_T_ maintained only a little less or about the same number of alleles.

The molecular inbreeding coefficient (*F*) was better restrained by maxA_T_, whereas maxK produced the highest molecular inbreeding levels, close to those yielded by RND. Method maxA_T_ was also the optimizing method making a wider use of the individuals available for mating (nMates) and produced the lowest variance of contributions from females to progeny (VarContFem), thus approaching the equalization of contributions from parents to progeny. Method maxH_T_ produced the highest variance of contributions from females to the progeny in the initial generations.

As already observed in previous studies, maxH_T_ tends to equalize allele frequencies within loci to reach the maximum possible heterozygosity. This can be seen as a reduction in the variance of allelic frequencies within loci (VarFreq). Method maxK was producing the largest variation in allelic frequencies.

All management methods involved an average number of one migrant per generation and subpopulation. However, there were differences in the variance of the number of migrants per subpopulation depending on the generations and methods (see graphs VarMigrants in **Figures 2** and **3**). The highest variation occurred in the initial generations when differences in diversity between subpopulations were larger. Most migrations in these initial generations occurred from the first subpopulation (that with the largest ancestral size; **Figure 1**) to the others (not shown). Method maxA_T_ was the optimizing procedure with the lowest variation in subpopulation migrations.

**Figure 4:**
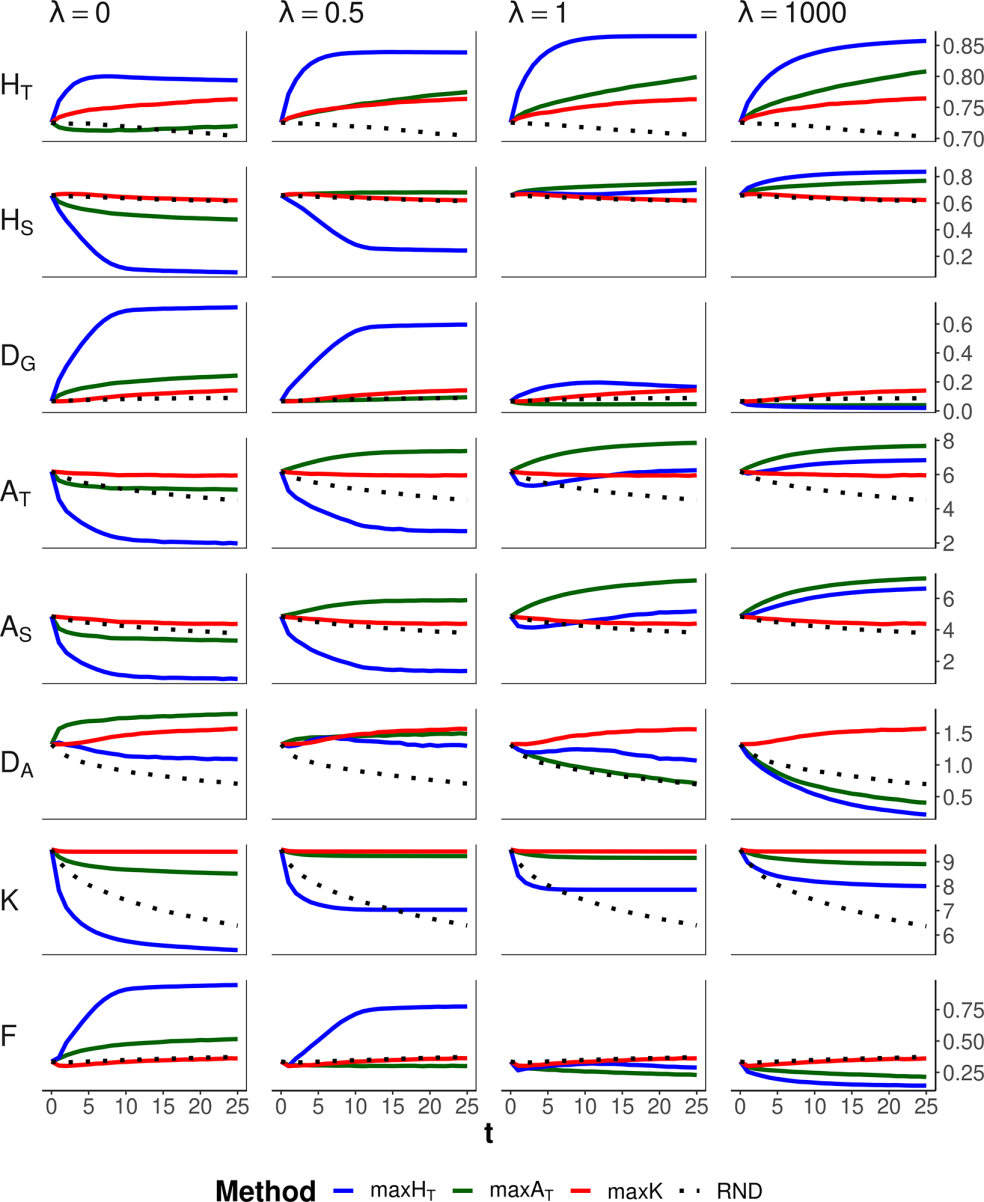
Changes in different diversity parameters over generations (*t*) in a subdivided population subjected to three optimization methods, maxH_T_ (blue line), maxA_T_ (green line), and maxK (red line), and an unmanaged control (RND, dotted black line). Optimization was made for a particular set of loci (12 multiallelic single-nucleotide polymorphism haplotype markers). In the case of maxH_T_ and maxA_T_, different between-population weighting factors (ခ*λ*) were assumed. One migrant per subpopulation and generation was considered in the optimizations. Statistics refer to the managed markers: total heterozygosity (*H* *_T_*) and its within- and between-subpopulation components (*H* *_S_* and *D* *_G_*); total allelic diversity (*A* *_T_*) and its within- and between-subpopulation components (*A* *_S_* and *D* *_A_*); total number of alleles in the population (*K*); and average coefficient of molecular inbreeding of individuals (*F*). Standard errors for means are lower than 0.01 (allelic measures), 0.001 (heterozygosity measures), and 0.002 (*F*).

**Figure 4** shows the results corresponding to the scenario of conservation of diversity for a particular set of loci (the same as in **Figure 2**) for a range of values of the weight (ခ*λ*) given to the within-subpopulation component. Method maxK and no management (RND) obviously were unaffected by the different weighting. Method maxH_T_ maintained the highest *H* *_T_* for all *λ* values. This was attained by increasing *D* *_G_* when all weight is given to the between-subpopulation component (ခ*λ* = 0) at the expense of decreasing the within-subpopulation component *H* *_S_*, or by increasing *H* *_S_* when all weight is given to the within-subpopulation component (ခ*λ* = 1,000) at the expense of decreasing the between-subpopulation component *D* *_G_*. Method maxA_T_ preserved better *A* *_T_* when some substantial weight was given to the within-subpopulation component (*i.e.,* λ ≥ 0.5). If all weight is given to the within-subpopulation component (ခ*λ* = 1,000), maxH_T_ would produce the lowest molecular inbreeding (*F*), as expected, but the number of alleles maintained would be lower than those obtained by the allelic optimization methods. For intermediate values of *λ* (0.5 or 1), maxA_T_ seems to be the most robust method, producing the lowest inbreeding and a number of alleles almost as large as that maintained by maxK, although giving lower *H* *_T_* than that of maxH_T_.

Additional simulations regarding alternative parameter settings with respect to those considered above are given as Supplementary figures. First, the results shown in **Figures 2**–**4** involved an average of one migrant per generation and subpopulation in the management period. **Supplementary Figure S1** presents results analogous to those of **Figure 2** but including a lower (0.4) and a higher (2) average number of migrations per subpopulation and generation, showing that the main results basically hold. Finally, **Figures 2**–**4** refer to simulations with a recombination rate between nucleotides of *c* = 10^–6^. **Supplementary Figures S2** and **S3** show results analogous to those of **Figures 2** and **3** but considering a recombination rate one order of magnitude larger (*c* = 10^–5^). The results are, in general terms, also similar to those obtained before.

## Discussion

Preservation of genetic diversity is one of the main objectives of conservation programs (Frankham et al., 2010; Allendorf et al., 2013; Oldenbroek, 2017). Because many threatened species have fragmented habitats and many populations maintained in captivity are structured, conservation methods should consider population subdivision and focus on a global management, including possible migrations among subpopulations, rather than being restricted to local efforts (Frankham et al., 2010, Chap. 17). In addition, in the absence of genealogical data, molecular markers are used to analyze population diversity and make conservation designs regarding genetic objectives (Benestan et al., 2016; Fuentes-Pardo and Ruzzante, 2017). Here we have addressed the question of which marker diversity parameters should be better considered for making conservation management decisions in a subdivided population. For multiallelic markers (such as microsatellite loci) or biallelic ones (such as SNPs) that can be analyzed as multiallelic ones if considering multi-SNP haplotypes (*e.g.,* Zhao et al., 2019), decisions can be taken on expected heterozygosity or allelic diversity measures. We have investigated the outcome of a subdivided population maintained with different optimization procedures aimed at maximizing heterozygosity or allelic diversity. Each method was successful in maintaining the diversity measure aimed at, but they showed remarkable differences on how much of the rest of diversity parameters are conserved, the distribution patterns of diversity within and between populations, and the level of molecular inbreeding (homozygosity). The results confirm some preliminary runs carried out by López-Cortegano et al. (2019) to illustrate the use of the software Metapop2 with multiallelic markers. Thus, allelic diversity methods, in particular maxA_T_, can be recommended as the method of choice because it maintains a high allelic richness in the population (uniformly distributed across subpopulations) and controls inbreeding rather efficiently.

We considered two scenarios regarding the number of markers to be managed. One in which a specific set of loci is the target for conservation, as it could apply, for example, to specific loci of interest, such as those related to the immune system. In this case, because management is carried out on the specific loci of interest, the management methods are very effective in increasing genetic diversity (**Figure 2**). Another scenario has the objective of preserving the whole genomic diversity by using a restricted number of markers. In this case, the management methods are obviously less effective (**Figure 3**), and the degree of success will depend on the number of markers considered and the genetic structure of the species. We used a relatively high density of markers and, in this situation, the methods were rather effective in conserving genetic diversity for the whole sequence. However, it is expected that the availability of only a low number of markers will be less effective in achieving proper management of the whole genome.

It has been suggested that the number of alleles relates more strongly to the long-term capacity of populations to adapt to changing environments (James, 1970; Hill and Rasbash, 1986; Wilson et al., 2009; Medugorac et al., 2011). Caballero and García-Dorado (2013) showed, through computer simulations, that the long-term adaptive potential of a subdivided population subject to natural selection relates more strongly to allelic diversity. Vilas et al. (2015) performed an experiment with *Drosophila melanogaster* in which synthetic populations were built from a group of subpopulations by maximizing either the heterozygosity or the total number of alleles for nine microsatellite loci. Artificial selection for sternopleural bristle number during eight generations showed that the response to selection was larger (for both upward and downward number of bristles) for synthetic populations obtained by maximizing the number of marker alleles than for those obtained by maximizing marker heterozygosity. In addition, it has been observed in *Arabidopsis halleri* that genome-wide SNP diversity does not show a significant correlation with microsatellite heterozygosity based on 20 markers but is significantly correlated with microsatellite allelic richness (Fischer et al., 2017). These results thus suggest that maximization of allelic diversity can be a more desirable conservation strategy than maximization of expected heterozygosity of multiallelic markers regarding the maintenance of the adaptive potential of populations. On the other hand, inbreeding must also be avoided because of the negative effects associated to inbreeding depression (Charlesworth and Willis, 2009). Method maxA_T_ seems to accomplish both objectives.

Maximizing global heterozygosity is achieved by leading genes to intermediate allele frequencies (Fernández et al., 2004). In fact, maximizing heterozygosity is equivalent to maximizing the effective number of alleles, that is, the number of alleles per locus if all had the same frequency (Crow and Kimura, 1970). We checked this by performing simulations where a global optimization is made on the total effective number of alleles in the population, finding results identical to those for maxH_T_ with *λ* = 1. In a single undivided population, this tendency for equalizing allelic frequencies within each locus has the advantage of leading rare alleles to intermediate frequencies and thus also avoiding their loss. Thus, in undivided populations, maxH_T_ can be the most appropriate method to be carried out for conserving both a high heterozygosity and a high number of alleles (Fernández et al., 2004). In subdivided populations, maxH_T_ also implies a reduction in the variance of allelic frequencies within loci (VarFreq in **Figures 2** and **3**), but maximization of global heterozygosity is made at the cost of an increase of homozygosity (and thus inbreeding) in each subpopulation, at least in the short term, and a substantial loss of alleles (*F* and *K*, respectively, in **Figures 2** and **3**). Only if all weight in the optimization is given to within-subpopulation variation, maxH_T_ would make the best control of molecular inbreeding (ခ*λ* = 1,000 in **Figure 4**). However, the overall number of alleles maintained would still be lower than that maintained by the allelic diversity optimization methods (*K* in **Figure 4**).

Regarding allelic diversity procedures, we have compared the allelic diversity partition suggested by Caballero and Rodríguez-Ramilo (2010) (maxA_T_) with a method aimed at maintaining the overall number of alleles in the population (maxK). Although the former can be used to control the distribution of allelic variants within and between subpopulations, the second is applied without such a control. A notable different outcome is observed with each method. Method maxK maximizes, as expected, the total number of alleles in the whole population, but alleles are distributed variably across subpopulations, as indicated by a high value of *D* *_A_*. In contrast, maxA_T_ maintains almost as many alleles as maxK in the whole population but keeps them more homogeneously distributed over subpopulations, as indicated by a low value of *D* *_A_*. In conservation programs of structured populations, the objective may be to maintain reservoirs of variation such that there is little overlap between different subpopulations, for example, when there are local adaptations and a risk of outbreeding depression, in which case, a method such as maxK could be more appropriate. However, the loss of a subpopulation implies, in this case, the irreversible loss of allelic variation. If, on the contrary, allelic diversity is maintained uniformly in all subpopulations, as achieved by maxA_T_ (and, to some extent, by maxH_T_), the loss of a subpopulation does not imply a loss of allelic diversity because each subpopulation would provide a backup for the others. In a recent article, Ramljak et al. (2018) have proposed to use the statistic *A* *_T_* to prioritize different European cattle breeds for conservation.

Ollivier and Foulley (2013) have argued that the partition of allelic diversity proposed by Caballero and Rodríguez-Ramilo (2010) does not meet two properties. First, that the partition of within- and between-subpopulation components is not orthogonal because both components are not independent. Second, that it does not meet concavity, which means that diversity cannot decrease when a subpopulation is added or increase when a subpopulation is dropped. The lack of these supposedly desirable properties also affects Nei’s heterozygosity partition because both partitions follow the same approach. The lack of orthogonality of Nei’s partition has also been discussed by Jost (2008) (but see also Whitlock, 2011; Wang, 2012). Ollivier and Foulley (2013) recommended a definition of allelic diversity that relies mostly on the presence of private alleles, that is, a subpopulation only contributes to the total allelic diversity if it carries unique alleles in the population. Thus, if the subpopulations have no private alleles, their contribution to global allelic diversity is null and, in that scenario, the distribution of the allelic variants across subpopulations is irrelevant. In that sense, method maxK, whose objective is to maximize the total number of allelic variants in the whole population, would be consistent with that view of managing allelic diversity. Our results, in fact, show that maxK maximizes the total number of alleles, but maxA_T_ produces almost the same outcome in terms of total allelic number with the desirable addition of a better control of inbreeding.

In summary, our results suggest that maxA_T_, the maximization of the total allelic diversity (*A* *_T_*) following Caballero and Rodríguez-Ramilo (2010), which represents the total number of alleles present in a given pair of subpopulations averaged for all possible pairs, could be recommended as a standard management method for conservation programs of structured populations on the basis that it is efficient in preserving allelic diversity, within-subpopulation variation, and restraining inbreeding, thus guaranteeing the capacity of adaptation to short- and long-term environmental challenges.

## Data Availability

The pipeline required to simulate all populations assayed as well as to run the corresponding population analyses and management simulations is available in https://gitlab.com/elcortegano/hadopt.

## Author Contributions

AC, EL-C, and JF contributed to the conception and design of the study; EL-C and AP-F developed the required software and pipelines; EL-C, RP, and AL performed computer simulations; AC and EL-C wrote the manuscript. All authors contributed to manuscript revision and read and approved the submitted version.

## Funding

This work was supported by Agencia Estatal de Investigación (AEI) (CGL2016-75904-C2), Xunta de Galicia (ED431C 2016-037), and Fondos Feder: “Unha maneira de facer Europa.” UVigo Marine Research Centre (CIM-UVIGO) is funded by the “Excellence in Research (INUGA)” Program from the Regional Council of Culture, Education and Universities, with co-funding from the European Union through the ERDF Operational Program Galicia 2014-2020.

## Conflict of Interest Statement

The authors declare that the research was conducted in the absence of any commercial or financial relationships that could be construed as a potential conflict of interest.

## References

[B1] Allendorf F. W. Luikart G. H. Aitken S. N. (2013). Conservation and the genetics of populations. Chichester, West Sussex, UK: John Wiley and Sons.

[B2] Ballou J. D. Lacy R. C. (1995). “Identifying genetically important individuals for management of genetic diversity in pedigreed populations,” in Population Management for Survival and Recovery. Eds. Ballou J. D. Gilpin M. Foose T. J. (New York: Columbia University Press), 76–111.

[B3] Bashalkhanov S. Pandey M. Rajora O. P. (2009). A simple method for estimating genetic diversity in large populations from finite sample sizes. BMC Genet. 10, 84. 10.1186/1471-2156-10-84 20003542 PMC2800116

[B4] Bataillon T. M. David J. L. Schoen D. J. (1996). Neutral genetic markers and conservation genetics: simulated germplasm collections. Genetics 144, 409–417. 8878704 10.1093/genetics/144.1.409 PMC1207513

[B5] Belkhir K. Dawson K. J. Bonhomme F. (2006). A comparison of rarefaction and Bayesian methods for predicting the allelic richness of future samples on the basis of currently available samples. J. Hered. 97, 483–492. 10.1093/jhered/esl030 16987938

[B6] Benestan L. M. Ferchaud A. L. Hohenlohe P. A. Garner B. A. Naylor G. J. P. Baums I. B. (2016). Conservation genomics of natural and managed populations: building a conceptual and practical framework. Mol. Ecol. 25, 2967–2977. 10.1111/mec.13647 27086132

[B7] Bennewitz J. Meuwissen T. H. E. (2006). Breed conservation priorities derived from contributions to the total future genetic variance. Proc. 8th World Congress on Genetics Applied to Livestock Production, CD-Rom Communication No. 9, 33–06.

[B8] Caballero A. García-Dorado A. (2013). Allelic diversity and its implications for the rate of adaptation. Genetics 195, 1373–1384. 10.1534/genetics.113.158410 24121776 PMC3832279

[B9] Caballero A. Rodríguez-Ramilo S. T. (2010). A new method for the partition of allelic diversity within and between subpopulations. Conserv. Genet. 11, 2219–2229. 10.1007/s10592-010-0107-7

[B10] Caballero A. Toro M. A. (2000). Interrelations between effective population size and other pedigree tools for the management of conserved populations. Genet. Res. 75, 331–343. 10.1017/S0016672399004449 10893869

[B11] Caballero A. Toro M. A. (2002). Analysis of genetic diversity for the management of conserved subdivided populations. Conserv. Genet. 3, 289–299. 10.1023/A:1019956205473

[B12] Charlesworth D. Willis J. H. (2009). The genetics of inbreeding depression. Nat. Rev. Genet. 10, 783–796. 10.1038/nrg2664 19834483

[B13] Crow J. F. Kimura M. (1970). An Introduction to Population Genetics Theory. New York: Harper & Row.

[B14] Falconer D. S. Mackay T. C. (1996). Introduction to Quantitative Genetics. 4th edn Harlow, Essex, UK: Longmans Green.

[B15] Fernández J. Toro M. A. Caballero A. (2004). Managing individuals’ contributions to maximize the allelic diversity maintained in small, conserved populations. Conserv. Biol. 18 (5), 1358–1367. 10.1111/j.1523-1739.2004.00341.x

[B16] Fernández J. Toro M. A. Caballero A. (2008). Management of subdivided populations in conservation programs: Development of a novel dynamic system. Genetics 179, 683–692. 10.1534/genetics.107.083816 18493080 PMC2390643

[B17] Fischer M. C. Rellstab C. Leuzinger M. Roumet M. Gugerli F. Shimizu K. K. (2017). Estimating genomic diversity and population differentiation - an empirical comparison of microsatellite and SNP variation in *Arabidopsis halleri*. BMC Genom. 18, 69. 10.1186/s12864-016-3459-7 PMC5225627 28077077

[B18] Frankham R. Ballou J. D. Briscoe D. A. (2010). Introduction to Conservation Genetics. Cambridge, UK: Cambridge university press. 10.1017/CBO9780511809002

[B19] Fuentes-Pardo A. P. Ruzzante D. E. (2017). Whole-genome sequencing approaches for conservation biology: Advantages, limitations and practical recommendations. Mol. Ecol. 26, 5369–5406. 10.1111/mec.14264 28746784

[B20] Haller B. C. Messer P. W. (2018). SLiM 3: forward genetic simulations beyond the Wright-Fisher model. Mol. Biol. Evol. 36 (3), 632–637. 10.1101/418657 PMC6389312 30517680

[B21] Hill W. G. Rasbash J. (1986). Models of long term artificial selection in finite populations. Genet. Res. 48, 41–50. 10.1017/S0016672300024642 3781245

[B22] James J. W. (1970). The founder effect and response to artificial selection. Genet. Res. 16, 241–250. 10.1017/S0016672300002500 5512250

[B23] Jost L. (2008). G_ST_ and its relatives do not measure differentiation. Mol. Ecol. 17, 4015–4026. 10.1111/j.1365-294X.2008.03887.x 19238703

[B24] Jost L. Archer F. Flanagan S. Gaggiotti O. Hoban S. Latch E. (2017). Differentiation measures for conservation genetics. Evol. Appl. 11, 1139–1148. 10.1111/eva.12590 PMC6050183 30026802

[B25] Kirk H. Freeland J. R. (2011). Applications and implications of neutral versus non-neutral markers in molecular ecology. Int. J. Mol. Sci. 12, 3966–3988. 10.3390/ijms12063966 21747718 PMC3131602

[B26] Leberg P. L. (2002). Estimating allelic richness: effects of sample size and bottlenecks. Mol. Ecol. 11, 2445–2449. 10.1046/j.1365-294X.2002.01612.x 12406254

[B27] López-Cortegano E. Pérez-Figueroa A. Caballero A. (2019). Metapop2: re-implementation of software for the analysis and management of subdivided populations using gene and allelic diversity. Mol. Ecol. Resour. 19, 1095–1100. 10.1111/1755-0998.13015 30938911

[B28] Luikart G. Allendorf F. Cornuet J. M. Sherwin W. (1998). Distortion of allele frequency distributions provides a test for recent population bottlenecks. J. Hered. 89, 238–247. 10.1093/jhered/89.3.238 9656466

[B29] Medugorac I. Veit-Kensch C. E. Ramljak J. Brka M. Markovic B. Stojanovic S. (2011). Conservation priorities of genetic in domesticated metapopulations: a study in taurine cattle breeds. Ecol. Evol. 1, 408–420. 10.1002/ece3.39 22393510 PMC3287311

[B30] Meuwissen T. H. E. (2007). “Operation of conservation schemes,” in Utilisation and Conservation of Farm Animal Genetic Resources. Ed. Oldenbroek K. (Wageningen, The Netherlands: Wageningen Academic Publishers), 167–193.

[B31] Mills L. S. Allendorf F. W. (1996). The one-migrant-per-generation rule in conservation and management. Conserv. Biol. 10, 1509–1518. 10.1046/j.1523-1739.1996.10061509.x

[B32] Nei M. (1973). Analysis of gene diversity in subdivided populations. Proc. Natl. Acad. Sci. U.S.A. 70, 3321–3323. 10.1073/pnas.70.12.3321 4519626 PMC427228

[B33] Oldenbroek J. K., editors. (2017). Genomic management of animal genetic diversity. The Netherlands: Wageningen Academic Publishers. 10.3920/978-90-8686-850-6

[B34] Ollivier L. Foulley J.-L. (2013). A note on the partition of allelic diversity. Conserv. Genet. 14, 1285–1290. 10.1007/s10592-013-0508-5

[B35] Petit R. J. El Mousadik A. Pons O. (1998). Identifying populations for conservation on the basis of genetic markers. Conserv. Biol. 12, 844–855. 10.1046/j.1523-1739.1998.96489.x

[B36] Ramljak J. Bunevski G. Bytyqi H. Marković B. Brka M. Ivanković A. (2018). Conservation of a domestic metapopulation structured into related and partly admixed strains. Mol. Ecol. 27, 1633–1650. 10.1111/mec.14555 29575253

[B37] Simianer H. (2005). Using expected allele number as objective function to design between and within breed conservation of farm animal biodiversity. J. Anim. Breed Genet. 122, 177–187. 10.1111/j.1439-0388.2005.00523.x 16130469

[B38] Steffen W. Richardson K. Rockström J. Cornell S. E. Fetzer I. Bennett E. M. (2015). Sustainability. Planetary boundaries: guiding human development on a changing planet. Science 347 (6223), 1259855. 10.1126/science.1259855 25592418

[B39] Szpiech Z. A. Jakobsson M. Rosenberg N. A. (2008). ADZE: a rarefaction approach for counting alleles private to combinations of populations. Bioinformatics 24, 2498–2504. 10.1093/bioinformatics/btn478 18779233 PMC2732282

[B40] Toro M. A. Fernández J. Caballero A. (2009). Molecular characterization of breeds and its use in conservation. Livest. Sci. 120, 174–195. 10.1016/j.livsci.2008.07.003

[B41] Toro M. A. Pérez-Enciso M. (1990). Optimization of selection response. Genet. Sel. Evol. 22, 93–107. 10.1186/1297-9686-22-1-93

[B42] Vales-Alonso J. Fernández J. González-Castaño F. J. Caballero A. (2003). A parallel optimization approach for controlling allele diversity in conservation schemes. Math. Biosci. 183, 161–173. 10.1016/S0025-5564(03)00037-3 12711409

[B43] Vilas A. Pérez-Figueroa A. Quesada H. Caballero A. (2015). Allelic diversity for neutral markers retains a higher adaptive potential for quantitative traits than expected heterozygosity. Mol. Ecol. 24 (7), 4419–4432. 10.1111/mec.13334 26222582

[B44] Wang J. (2012). On the measurements of genetic differentiation among populations. Genet. Res. 94, 275–289. 10.1017/S0016672312000481 23298450

[B45] Weiser E. L. Grueber C. E. Jamieson I. G. (2012). AlleleRetain: a program to assess management options for conserving allelic diversity in small, isolated populations. Mol. Ecol. Resour. 12, 1083–1091. 10.1111/j.1755-0998.2012.03176.x 22925629

[B46] Whitlock M. C. (2011). *G´* *_ST_* and *D* do not replace *F* . Mol. Ecol. 20, 1083–1091. 10.1111/j.1365-294X.2010.04996.x 21375616

[B47] Wilson A. Arcese P. Keller L. F. Pruett C. L. Winker K. Patten M. A. (2009). The contribution to island populations to *in situ* genetic conservation. Conserv. Genet. 10, 419–430. 10.1007/s10592-008-9612-3

[B48] Wright S. (1952). The theoretical variance within and among subdivisions of a population that is in a steady state. Genetics 37, 312–321. 17247394 10.1093/genetics/37.3.312 PMC1209559

[B49] Zhang M. Peng W. F. Hu X. J. Zhao Y. X. Yang J. (2018). Global genomic diversity and conservation priorities for domestic animals are associated with the economies of their regions of origin. Sci. Rep. 8 (1), 11677. 10.1038/s41598-018-30061-0 30076315 PMC6076285

[B50] Zhao Q.-B. Sun H. Zhang Z. Xu Z. Olasege B. S. Ma P.-P. (2019). Exploring the structure of haplotype blocks and genetic diversity in Chinese indigenous pig populations for conservation purpose. Evol. Bioinform. Online 15, 1176934318825082 10.1177/1176934318825082. PMC6348539 30718942

