# Supplementary material for: Optimal Management of Genetic Diversity in Subdivided Populations
Source: Front Genet. 2019 Sep 13;10:843. doi: 10.3389/fgene.2019.00843 (PMC6753960; doi:10.3389/fgene.2019.00843)

Figure S1. Changes in different diversity parameters over generations (t) in a subdivided population subjected to three optimization methods: maxH_T (blue line), maxA_T (green line), and maxK (red line), and an unmanaged control (RND, dotted black line). Optimization was made for 12 multiallelic (SNP haplotype) markers. In the case of maxH_T and maxA_T, a between-population weighting factor of $\lambda = 1$ (*i.e.* equal weight for within and between-subpopulation components of diversity) was assumed. An average of 0.4 or 2 migrants (mig) per subpopulation and generation were considered in the optimizations. Statistics measured in the managed markers: Total heterozygosity (H_T); Total allelic diversity (A_T); Total number of alleles in the population (K); Average coefficient of molecular inbreeding of individuals (F). Standard errors for means are lower than 0.011 (A_T and K), 0.0006 (H_T) and 0.002 (F).

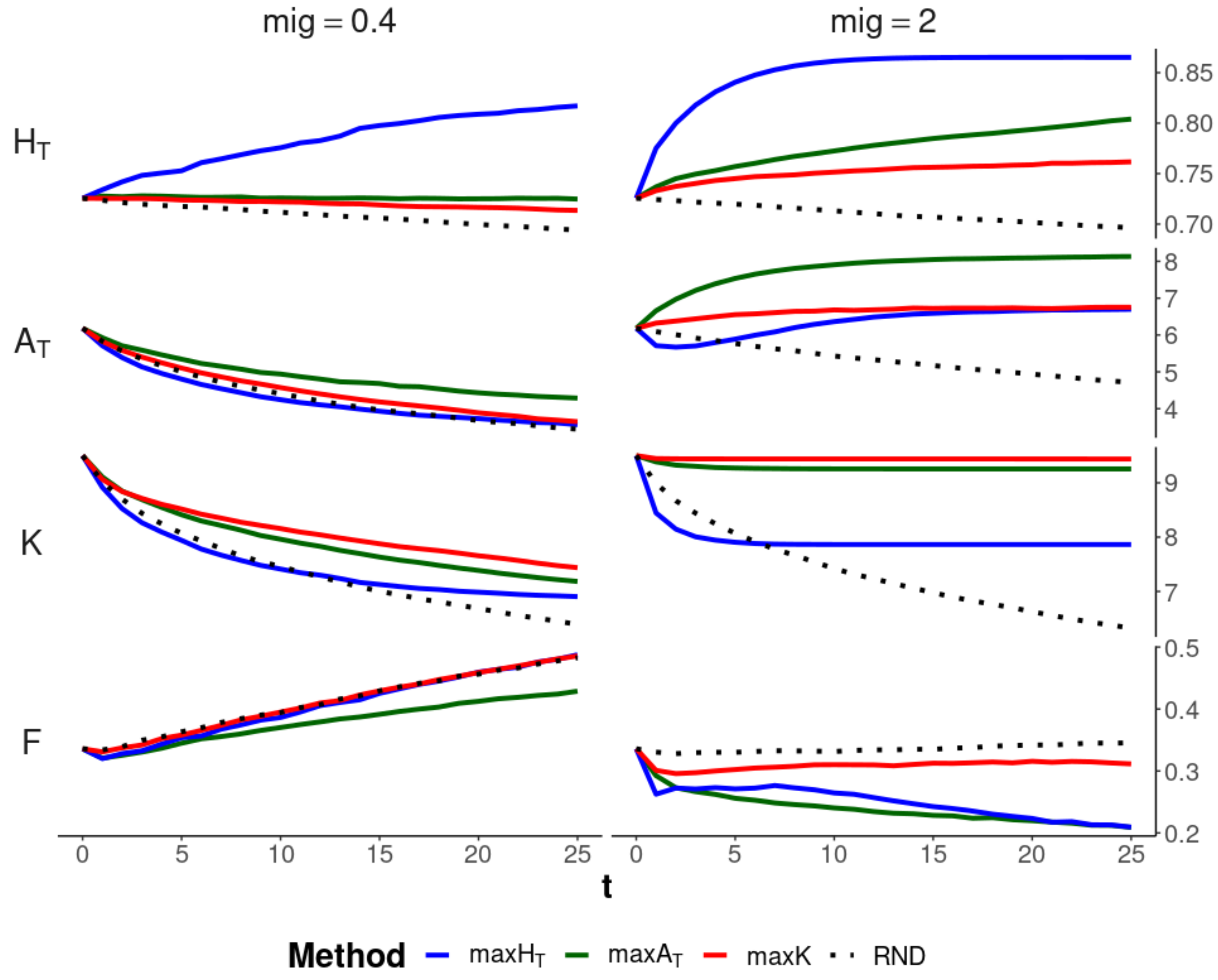

Supplement: Supplementary file 1 [file Image_1.pdf]
