# Supplementary material for: Optimal Management of Genetic Diversity in Subdivided Populations
Source: Front Genet. 2019 Sep 13;10:843. doi: 10.3389/fgene.2019.00843 (PMC6753960; doi:10.3389/fgene.2019.00843)

Figure S2. Changes in different diversity parameters over generations (*t*) in a subdivided population subjected to three optimization methods: maxH_T (blue line), maxA_T (green line), and maxK (red line), and an unmanaged control (RND, dotted black line). Optimization was made for 12 multiallelic (SNP haplotype) markers. In the case of maxH_T and maxA_T, a between-population weighting factor of $\lambda = 1$ (*i.e.* equal weight for within and between-subpopulation components of diversity) was assumed. One migrant per subpopulation and generation was considered in the optimizations. The recombination rate assumed in the simulations is one order of magnitude higher that in Figure 2 of the main text ($c = 10^{-5}$). Statistics measured in the managed markers: Total heterozygosity (H_T) and its within and between-subpopulation components (H_S and D_G); Total allelic diversity (A_T) and its within and between-subpopulation components (A_S and D_A); Total number of alleles in the population (K); Average coefficient of molecular inbreeding of individuals (F); Number of pairing mates involved in the different procedures (nMates); Variance of the contribution from female parents to progeny (varContFem); Variance of allelic frequencies with loci (VarFreq); and Variance of the number of migrants per subpopulation (VarMigrants). Standard errors for means are lower than 0.01 (allelic measures), 0.0008 (heterozygosity measures), 0.002 (F).

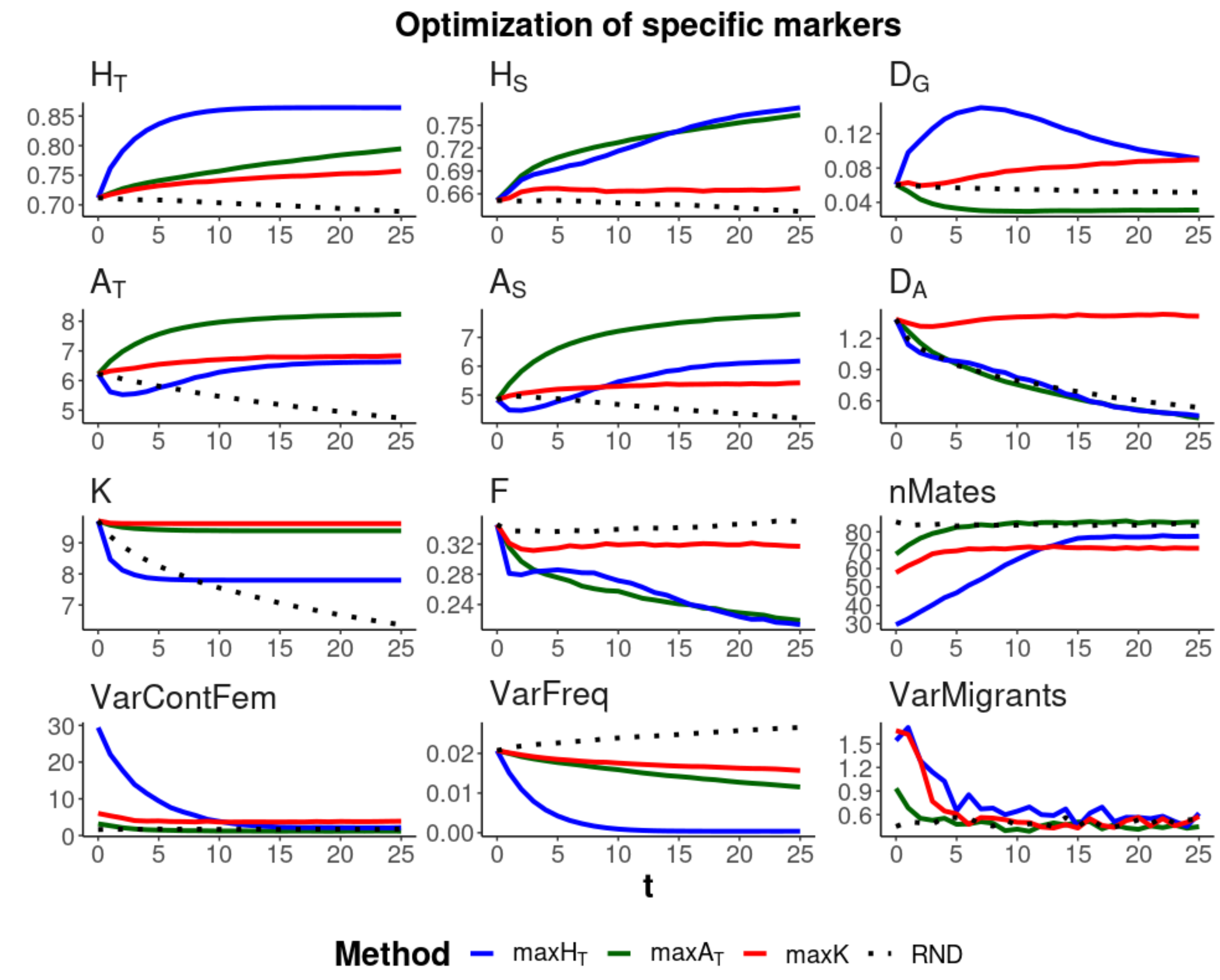

Supplement: Supplementary file 2 [file Image_2.pdf]
