# Supplementary material for: Optimal Management of Genetic Diversity in Subdivided Populations
Source: Front Genet. 2019 Sep 13;10:843. doi: 10.3389/fgene.2019.00843 (PMC6753960; doi:10.3389/fgene.2019.00843)

Figure S3. Changes in different diversity parameters over generations (*t*) in a subdivided population subjected to three optimization methods: maxH$_T$ (blue line), maxA$_T$ (green line), and maxK (red line), and an unmanaged control (RND, dotted black line). Optimization was made for 120 multiallelic (SNP haplotype) markers but statistics calculated for the whole simulated genome. Simulation characteristics and statistics as in Figure S2. The recombination rate assumed in the simulations is one order of magnitude higher that in Figure 2 of the main text ($c = 10^{-5}$). Standard errors for means are lower than 0.01 (allelic measures), 0.0008 (heterozygosity measures), 0.002 (F).

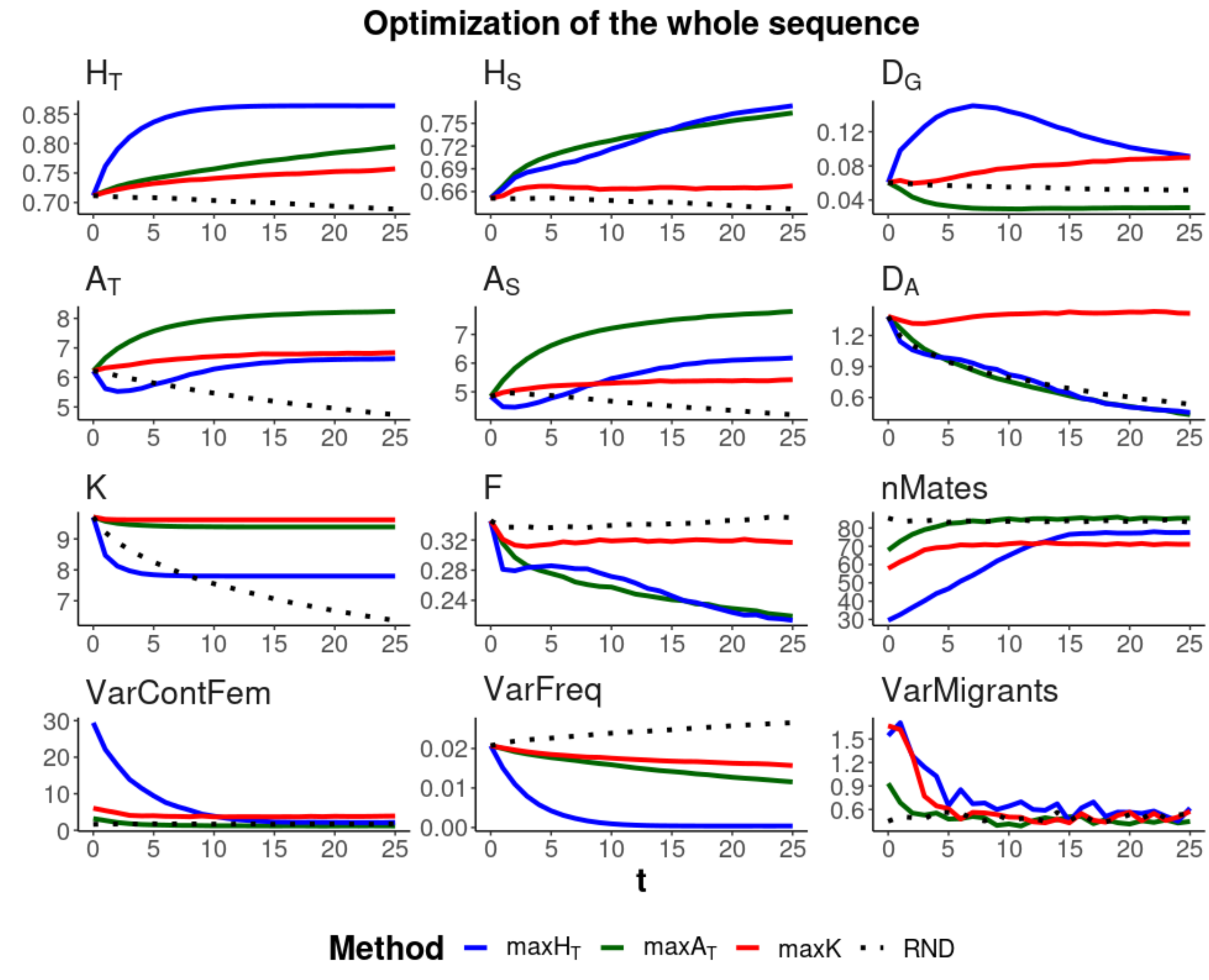

Supplement: Supplementary file 3 [file Image_3.pdf]
