# Supplementary material for: Optimal Management of Genetic Diversity in Subdivided Populations
Source: Front Genet. 2019 Sep 13;10:843. doi: 10.3389/fgene.2019.00843 (PMC6753960; doi:10.3389/fgene.2019.00843)

**SUPPLEMENTARY FIGURES**

Optimal management of genetic diversity in subdivided populations

Eugenio López-Cortegano, Ramón Pouso, Adriana Labrador, Andrés Pérez-Figueroa, Jesús Fernández, Armando Caballero

**Figure S1.** Changes in different diversity parameters over generations (*t*) in a subdivided population subjected to three optimization methods: maxH_T_ (blue line), maxA_T_ (green line), and maxK (red line), and an unmanaged control (RND, dotted black line). Optimization was made for 12 multiallelic (SNP haplotype) markers. In the case of maxH_T_ and maxA_T_, a between-population weighting factor of *λ* = 1 (*i.e.* equal weight for within and between-subpopulation components of diversity) was assumed. An average of 0.4 or 2 migrants (mig) per subpopulation and generation were considered in the optimizations. Statistics measured in the managed markers: Total heterozygosity (*H_T_*); Total allelic diversity (*A_T_*); Total number of alleles in the population (*K*); Average coefficient of molecular inbreeding of individuals (*F*). Standard errors for means are lower than 0.011 (*A_T_* and *K*), 0.0006 (*H_T_*) and 0.002 (*F*).


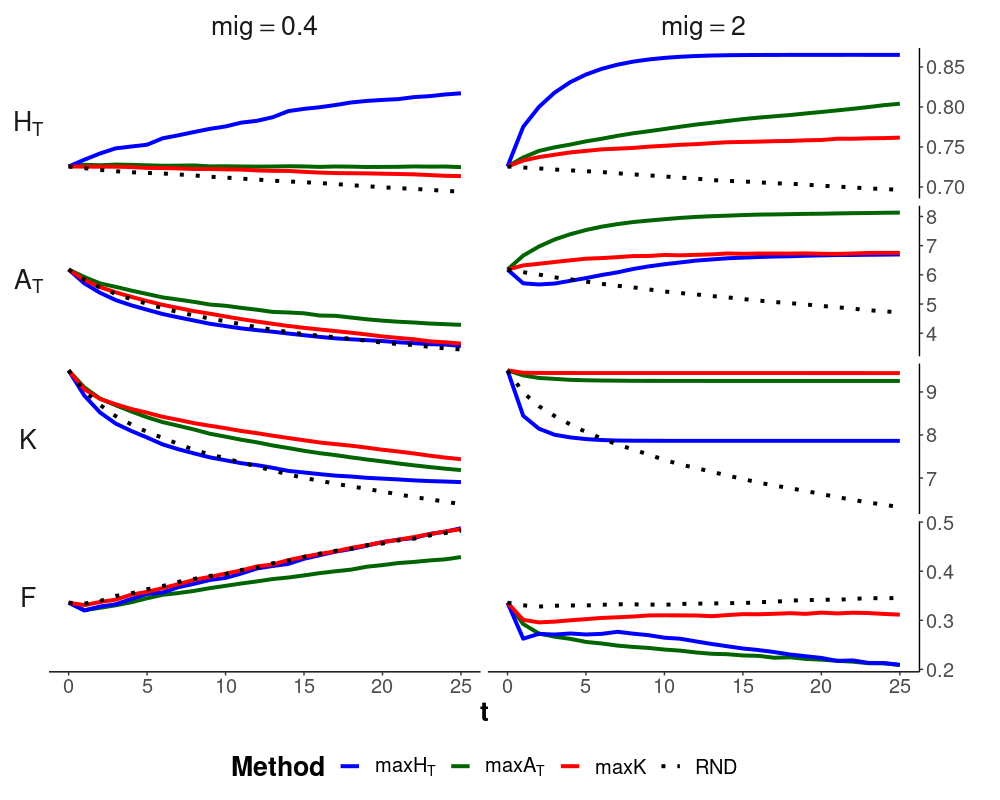


**Figure S2.** Changes in different diversity parameters over generations (*t*) in a subdivided population subjected to three optimization methods: maxH_T_ (blue line), maxA_T_ (green line), and maxK (red line), and an unmanaged control (RND, dotted black line). Optimization was made for 12 multiallelic (SNP haplotype) markers. In the case of maxH_T_ and maxA_T_, a between-population weighting factor of *λ* = 1 (*i.e.* equal weight for within and between-subpopulation components of diversity) was assumed. One migrant per subpopulation and generation was considered in the optimizations. The recombination rate assumed in the simulations is one order of magnitude higher that in Figure 2 of the main text (*c* = 10^-5^). Statistics measured in the managed markers: Total heterozygosity (*H_T_*) and its within and between-subpopulation components (*H_S_* and *D_G_*); Total allelic diversity (*A_T_*) and its within and between-subpopulation components (*A_S_* and *D_A_*); Total number of alleles in the population (*K*); Average coefficient of molecular inbreeding of individuals (*F*); Number of pairing mates involved in the different procedures (nMates); Variance of the contribution from female parents to progeny (varContFem); Variance of allelic frequencies with loci (VarFreq); and Variance of the number of migrants per subpopulation (VarMigrants). Standard errors for means are lower than 0.01 (allelic measures), 0.0008 (heterozygosity measures), 0.002 (*F*).


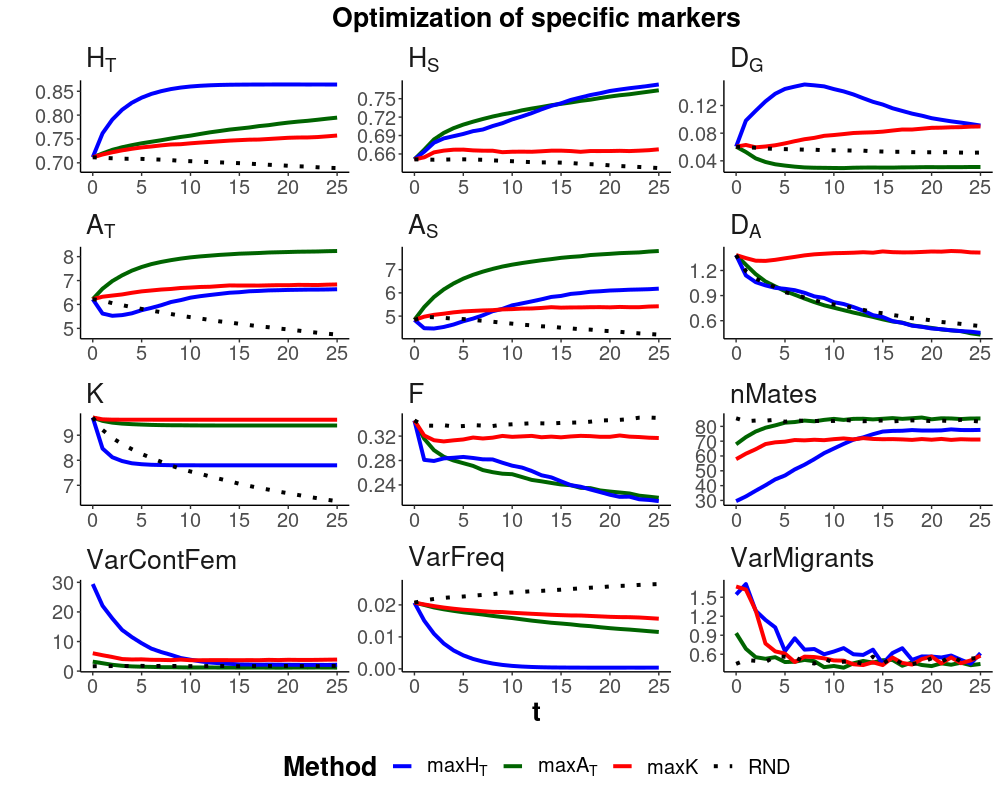


**Figure S3.** Changes in different diversity parameters over generations (*t*) in a subdivided population subjected to three optimization methods: maxH_T_ (blue line), maxA_T_ (green line), and maxK (red line), and an unmanaged control (RND, dotted black line). Optimization was made for 120 multiallelic (SNP haplotype) markers but statistics calculated for the whole simulated genome. Simulation characteristics and statistics as in Figure S2. The recombination rate assumed in the simulations is one order of magnitude higher that in Figure 2 of the main text (*c* = 10^-5^). Standard errors for means are lower than 0.01 (allelic measures), 0.0008 (heterozygosity measures), 0.002 (*F*).


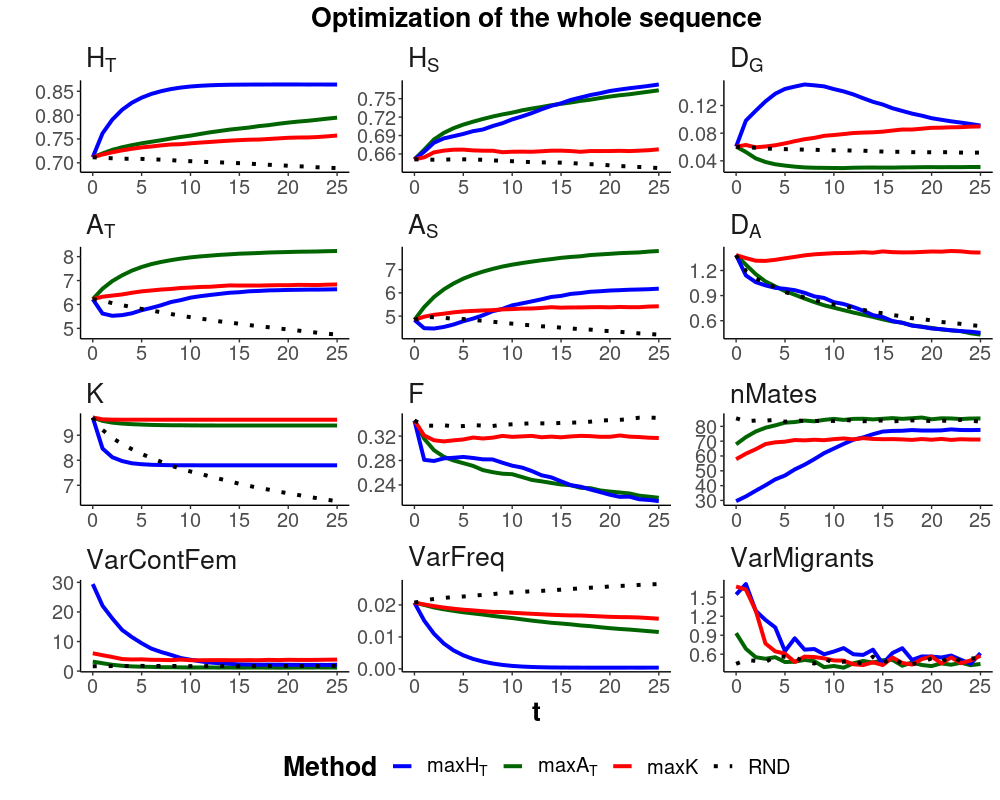

Supplement: Supplementary file 4 [file Table_1.docx]
